# Virus variant quantification with Orthanq

**DOI:** 10.1186/s12859-026-06387-2

**Published:** 2026-02-04

**Authors:** Hamdiye Uzuner, Felix Wiegand, Sven Schrinner, David Lähnemann, Dirk Schadendorf, Johannes Köster

**Affiliations:** 1 Bioinformatics and Computational Oncology, Institute for Artifical Intelligence in Medicine (IKIM), Faculty of Medicine, University Hospital Essen, University of Duisburg-Essen, Essen, Germany; 2 Department of Dermatology, University Hospital Essen, West German Cancer Center, University Duisburg-Essen, Essen, Germany; 3 German Cancer Consortium (DKTK), Partner Site Essen-Düsseldorf, A Partnership Between DKFZ and University Hospital Essen, Essen, Germany

**Keywords:** Haplotype quantification, SARS-CoV-2, Virus lineage quantification, Uncertainty-computation, Co-infection

## Abstract

**Background:**

Existing tools for virus variant identification can pinpoint the most abundant virus variant in a sequencing sample. However, patients can be infected by more than one variant of the same virus species or strain, for example by multiple variants of SARS-CoV-2. This leads to the more complicated problem of virus variant quantification from samples containing virus mixtures.

**Results:**

We report on improvements of Orthanq, our generic tool for haplotype quantification, and show how it can be applied to perform uncertainty aware quantification of virus variants. We evaluate this ability on simulated and real SARS-CoV-2 and HIV-1 virus mixture datasets and show that Orthanq outperforms other state of the art approaches.

**Conclusions:**

Orthanq performs identification and uncertainty-aware quantification of known virus variants of any virus species, in particular also in samples with mixed infections. With extensive built-in visualizations and reporting of alternative solutions with posterior densities, users can easily evaluate the uncertainty of the results.

## Background

Virus variants are full length viral genome sequences with only a small number of genomic variants in comparison to a consensus reference genome of a respective virus species, strain, lineage or subtype.^1^ Here, the distinction between virus variants and genomic variants is important: genomic variants are individual nucleotide sequence differences between a virus variant and the corresponding virus strain reference genome. Thus, the collection of all the genomic variants that a virus variant bears with respect to the reference genome, define the full haplotype of this virus variant genome from start to end. Therefore, data for virus variant identification needs to cover those genomic variants. Such data can be generated by targeting regions containing genomic variants with primers in an RT-PCR [1], with probes on a microarray chip [2], with primers for multiplexed amplicon-sequencing [3], or with hybrid-capture probes for all variants of a specific virus strain [4]. For virus variant identification from diverse and well-characterized virus species, this will usually require covering the whole viral genome with either of these technologies, as genomic variants will be found throughout the entire sequence length.

Tools for virus variant identification from such data draw on the full genome sequence of known virus variants.^2^ Two tools that are commonly used for virus variant identification from sequencing data in SARS-CoV-2 (severe acute respiratory syndrome coronavirus 2) are Pangolin [6] and Nextclade [7]. Pangolin employs a combination of decision trees and random forest models, orchestrated by a Snakemake [8] workflow. It predicts the most likely virus variant from a given sequence, which is typically obtained by assembly of second or third generation sequencing data. Nextclade uses Smith-Waterman alignment with reading-frame-dependent gap opening penalties to identify the closest clade to a given sequence assembly on a phylogenetic tree via an exhaustive search. But since the sequence assembly usually happens *de novo*, it does not draw on the prior knowledge of small genomic variants that distinguish closely related virus variants. As a result, such subtle differences are often eliminated and these tools can only capture the most abundant variant.

However, samples from a viral infection can be more complex, for example when co-infections cause a sample to contain multiple variants of the same virus. This is especially true for rapidly evolving viruses like SARS-CoV-2, a virus of the *Coronaviridae* family that emerged in late 2019. The number of characterized SARS-CoV-2 virus variants was already reported as more than 1300 in 2022 [9], with this number further increasing to about 3000 as of 2024 [10]. In accordance with this rapid evolution, several studies during the COVID-19 pandemic have shown the existence of co-infections by multiple SARS-CoV-2 variants [4, 11]. Thus, for comprehensive surveillance, we need to move from identifying only the most abundant virus variant in a sample, to reliably quantifying all of the virus variants that are present.

One quantitative approach that can be re-appropriated for virus variant identification, is RNA transcript quantification. For example, Thomas et al. [12] use Kallisto [13] for virus variant identification, a tool which performs a k-mer based pseudoalignment of reads against a set of known sequences while accounting for common and exclusive k-mers via equivalence classes. This allows quantifying multiple given virus variants (with known genome sequences) in a sample, instead of only making a single prediction for the most abundant one.

In this work, we also aim to quantify multiple (previously known) virus variants from a virus mixture and to do this in an uncertainty-aware manner. To this end, we extend our previously published tool Orthanq (Orthogonal evidence based HAplotype Quantification) [14], which we developed for uncertainty-aware haplotype quantification. Orthanq employs a Bayesian model to quantify any kind of haplotypes that can be registered as genomic variant differences compared to a given reference sequence. It uses a Bayesian model to obtain uncertainty aware predictions on top of posterior genomic variant allele frequency distributions provided by Varlociraptor [15]. In Uzuner et al. [14], we showed that Orthanq performs superior for Human Leukocyte Antigen (HLA) typing (quantifying the composition of HLA haplotypes in a given human sample). Here, we improve Orthanq’s model and output, to better quantify virus variants. For this purpose, each virus variant is considered a haplotype, with its genomic variants determined by comparison to a given reference genome sequence for the respective virus species or strain. We demonstrate its accuracy on a simulated dataset that mimics the progress of a pandemic, as well as two real datasets: one with 13 thoroughly characterized SARS-CoV-2 co-infection samples, and one with an *in vitro* mixture sample of 5 HIV-1 virus variants at known fractions.

## Methods

### Virus lineage quantification with Orthanq

**Fig. 1:**
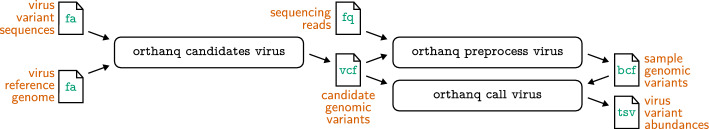
Overview of the three steps involved in virus variant quantification using Orthanq, with the command line calls required. Input and output file formats are given within document icons, with orange descriptions of file contents. File formats are: fa—FASTA sequence format, fq—FASTQ sequencing data with quality values, vcf—variant call format, bcf—binary version of vcf, tsv— tab-separated values

To apply Orthanq for quantification of virus variants we mostly follow the same approach that was used to apply Orthanq for HLA typing [14]. The practical implementation is depicted in Fig. 1.

In the following we briefly describe the approach, while highlighting the differences and improvements made in this study. Orthanq’s code and its development can be reviewed in its repository at https://github.com/orthanq/orthanq.

#### Constructing candidate genomic variants

Independent of the samples to be evaluated, Orthanq constructs a set of candidate genomic variants that comprehensively describe the differences of the virus variants (i.e. the haplotypes to quantify) and the respective virus reference genome. The first step is the alignment of the virus variant sequences to the virus reference genome with Minimap2 [16]. From the edit operations in the resulting alignments (represented as so-called CIGAR strings), we obtain individual genomic variants that represent the differences between the virus variant haplotypes and the reference genome. The resulting genomic variants are typically single nucleotide variants (SNVs) and small insertions and deletions (indels). We collect the union of these (candidate) genomic variants from all known virus variant haplotypes, and record the presence of each variant for each virus variant as a matrix in VCF format. The entire process is available as a subcommand (*orthanq candidates virus*) of Orthanq’s command line interface for easy integration into computational pipelines.

#### Sample preprocessing

For any sample to be quantified with Orthanq, one first has to obtain read alignments and, with that, obtain posterior allele frequency distributions of each candidate genomic variant as constructed above. While these steps can be conducted individually, Orthanq offers an all in one subcommand for this (*orthanq preprocess virus*).

Experience shows that read alignment works best when using a pangenome based read aligner that is able to consider all virus variants as alternative haplotypes, thus minimizing reference bias induced erroneous read placement [14]. For that reason, Orthanq uses vg autoindex [17] to generate a pangenome alignment index from a given candidate genome variant file as described above. Then, vg giraffe [18] is used to perform the read alignment.

When previously publishing the application of Orthanq for HLA typing [14], we used a two-step alignment strategy: we first aligned reads to a linear genome, then extracted reads mapping to HLA loci, which were subsequently aligned to a pangenome of all given HLA alleles. The reason for this two-step strategy was that it improves performance, since linear alignment can still be faster than pangenome alignment. Here, since all considered haplotypes cover the entire reference genome, the initial linear alignment can be skipped.

Varlociraptor [15] is then used to calculate posterior allele frequency distributions of each candidate genomic variants (in VCF format). The sensitivity of this approach, together with the fact that not only point estimates but the whole allele frequency distribution is inferred, is a major factor for being able to accurately quantify even lowly abundant virus variants and associated uncertainties.

#### Virus variant quantification

After preprocessing a sample, Orthanq quantifies the haplotypes given via the candidate genomic variant file (invoked by the subcommand *orthanq call virus*). As we previously described [14], this works via a two step process: (i) a linear optimization for finding a set of candidate haplotypes, and (ii) a Bayesian model for calculating a set of posterior primary and alternative haplotype compositions with associated posterior densities (thereby highlighting the uncertainty in the data). The reason for (i) is to prune the search space for the Bayesian model, the runtime complexity of which is exponential in the number of considered haplotypes. While the Bayesian model remains as previously published [14] (also briefly repeated at the end of this section), we have improved the linear optimization step in the following way.

The principle of the linear optimization remains as before: across all candidate genomic variants, it minimizes the distance between the sum of all haplotype fractions that carry a genomic variant allele, and the observed maximum a posteriori estimate of that genomic variant’s allele frequency (as obtained from Varlociraptor during the preprocessing). This is based on the idea that the sum of haplotype fractions explain the variants and their allele frequencies observed in the data.

Let $H = \{h_1, h_2,..., h_n\}$ be the set of considered haplotypes (i.e. here, the virus variants), let $\{v_1, v_2,..., v_k\}$ be the union of all genomic variants of those haplotypes, and let

$$ \left( V_{i,j} \right) _{i = 1,2,\ldots ,n,j = 1,2,\ldots ,k} $$

be the matrix denoting whether haplotype $h_i$ has variant $v_j$. Further, let $\widehat{\theta _j}$ be the maximum a posteriori allele frequency of genomic variant $v_j$ and let $\psi _i$ denote the latent fraction of haplotype $h_i$.

In this work, we additionally filter the set of haplotypes that are considered for the linear optimization to a set of representatives that exhibit a pairwise distance of at least one in terms of the genomic variants that are covered in the data. This avoids redundant computations in the linear optimization. Formally, we restrict the set of haplotypes *H* considered for the linear optimization to the subset $H' \subseteq H$ such that each pair of haplotypes $h, h'$ in $H'$ differs by at least one genomic variant. After the model evaluation, alternative haplotypes with the same set of genomic variants are re-added as alternative solutions with the same probability.

Further, we now weight each genomic variant by how certain Varlociraptor is about its presence or absence. In other words, the less evidence there is for a particular genomic variant, the less important it is for the linear optimization. Let $p_j$ be the posterior probability of the variant $v_j$ to be present and $a_j$ the one to be absent as reported by Varlociraptor. We define the weight $lambda_j$ for every genomic variant $v_j$ as

$$ \lambda = \max (p_{j},a_{j}). $$

that is, the most certain statement about either presence or absence of the variant reported by Varlociraptor. We use this weight to ensure that uncertain genomic variants contribute less to the optimization than ones where the evidence is very clear. Before, Orthanq used a fixed threshold instead, which could either be unnecessarily strict (thus wasting evidence), or be too lenient, such that uncertain genomic variants could collectively overrule certain ones.

The linear optimization is then updated as follows:

$$\begin{aligned} \min \quad&\sum _{j \in W} \lambda _j \cdot \left| \left( \sum _{h_i \in H'} \psi _i V_{i,j} \right) - \hat{\theta }_j \right| \\ \text {s.t.} \quad&\psi _i \in [0,1], \quad \forall h_i \in H' \\&\sum _{h_i \in H'} \psi _i = 1 \\&\sum _{h_i \in H'} \mathbf {1}_{\psi _i > 0} = C \end{aligned} $$

As before [14], $W \subseteq \{1, 2, \ldots , k\}$ is the subset of genomic variant indices that are covered by all haplotypes. The last constraint, which ensures that the linear optimization finds exactly *C* haplotypes with a fraction greater than zero, is new. This way, we ensure (a) that there are enough candidates for the Bayesian model to explore the uncertainty in the data and consider alternative solutions and (b) that there are not more candidate haplotypes than the model can handle without too severe performance impact. The number *C* can be configured at the command line interface of Orthanq (default is $C=5$, which should suffice for virus variant quantification as discussed in this work).

In practice, above indicator variable ($\mathbf {1}_{\psi _i > 0}$) is modelled in the following way. We first replace the constraint with one using binary surrogate variables $z_i \in \{0,1\}$ for each haplotype $h_i$, in other words

$$ \sum _{h_i \in H'} z_i = C $$

Then, $z_i$ is constrained such that it switches to 1 when $psi_i > 0$ and remains at 0 otherwise:

$$ z_i \ge \psi _i, \quad z_i \le M \cdot \psi _i $$

with *M* being chosen sufficiently large. With introduction of the binary variables to the formula, the original Integer Linear Program (ILP) is turned to a Mixed-Integer Linear Program (MILP). While this sacrifices the polynomial complexity of the optimization, we found this to have no practical impact on runtimes for all investigated problem sizes.

The set of haplotypes predicted by the linear optimization is extended by haplotypes that are equivalent under *W*. These are then evaluated via the full Bayesian model as presented before [14]. This takes the full set of genomic variants and the posterior allele frequency distributions reported by Varlociraptor into account and utilizes a uniform prior for the haplotype fractions $\psi _1, \psi _2,..., \psi _n$. As before, this yields the posterior distribution of haplotype fractions

$$ \psi _1, \psi _2, \ldots , \psi _n \mid \boldsymbol{Z} $$

with $\boldsymbol{Z}$ denoting the observed (sequenced) DNA fragments over all involved genomic variants *H*. We refer to the original publication for the (unchanged) formal details [14]. Finally, the obtained distribution is extended by all equivalent realizations of $\psi _1, \psi _2,..., \psi _n$ where haplotypes $h_i \in H'$ are replaced by any haplotypes $h' \in H \setminus H'$ (in all possible combinations) that have before been removed in favor of $h_i$ as representative with the same set of genomic variants (see above). By doing this after the model evaluation, we save computational time without sacrificing accuracy, since the respective haplotypes have exactly the same genomic variants.

### Evaluation

For evaluation, we compared Orthanq with three other tools, namely Pangolin (v4.3.1) [6], Nextclade (v3.9.1) [7] and Kallisto (v0.46.0) [13], on three different datasets.^3^ While Orthanq and Kallisto were applied to all datasets, Pangolin and Nextclade were only applicable to the SARS-CoV-2 co-infection dataset. The evaluation workflow, including the tool versions used, can be reviewed at https://github.com/orthanq/orthanq-evaluation-virus.

#### Tool parametrization and usage

Pangolin and Nextclade require assembled virus variant genomes in FASTA format. These were generated with the sequencing reads as input, by running the Uncovar pipeline [12]. We then used the following columns for retrieving pango lineage and WHO names: Nextclade_pango and clade_who for Nextclade; lineage and scorpio_call for Pangolin. For Pangolin, further note that it reported Omicron predictions for Probable Omicron (Unassigned), Omicron (Unassigned), and Omicron (BA.1-like); and delta predictions for Probable Delta (Unassigned), Delta (B.1.617.2-like) and Delta (AY.4-like).

Kallisto and Orthanq both require a collection of known virus variants of a virus species in FASTA format. For SARS-CoV-2, a collection of FASTA sequences of Pango lineages [10] (retrieval date: August 2024), a repository that is regularly being updated, was aligned to the SARS-CoV-2 reference genome (NC_045512.2).

For HIV-1, we obtained accession IDs from the LANL HIV Sequence Database^4^ using the following parameter selections: Alignment type to Super filtered web, organism to HIV-1/SIVcpz, Region to Genome, Subtype to M-group without recombinants (A-L), DNA/Protein to DNA, Year to 2022. By that, we strived to consider a realistic set of HIV strains, simulating a de-novo analysis where the virus variants in the investigated sample are unknown. This set of variants already contained three of the five strains considered by Giallonardo et al. [19], namely YU2 (M93258.1), HXB2 (K03455) & 896 (U39362.2). We extended the set to include the two remaining strains NL4-3 (AF324493.2) and JR-CSF (M38429.1).

For Kallisto, the set of virus variant sequences were used to build the index for sequence quantification. For SARS-CoV-2, after quantification, the 5 most abundant virus variants were considered as the predicted haplotypes. Their predicted fraction in the given sample was obtained by dividing the abundance by the total sum of reported abundances of all haplotypes. All other abundances except the five most abundant haplotypes were summed and summarized as "other". For HIV-1, abundances of each virus variant were calculated by dividing the abundance by the total sum of abundances.

For Orthanq, Fig. 1 shows the steps that were followed by applying Orthanq’s virus variant quantification related subcommands, using the obtained sequences in FASTA format and defining a species-specific reference genome, Wuhan-Hu-1 (NC_045512.2) for SARS-CoV-2 and HXB2 (K03455.1) for HIV-1.

All comparison tools were executed with their respective default settings.

### Datasets

#### SARS-CoV-2 simulated virus variant mixture

We created a simulated dataset, mimicking the progress of the SARS-CoV-2 pandemic during 2020–2025. Sequence counts of GISAID for USA were obtained from Nextstrain resources^5^ [20]. It contains weekly raw counts for each state, which we aggregated into nationwide weekly counts. From those summed up weekly case counts, we removed those below 50 and calculated frequencies for remaining virus variants for each week. We simulated two sets of reads for each virus variant using Mason [21]: one each with a coverage of 100x and 1000x. We used the following formula:

$$ \text {Number of reads} = \frac{\text {Coverage} \times \text {Genome size}}{\text {Read length}} $$

Then, we generated 10 samples for each week of the pandemic. For each of these 10 samples, we randomly selected three virus variants from the set of virus variants reported for that week. We then sampled simulated reads from these three virus variants in amounts proportional to their frequencies in the respective week, and combined these subsampled read sets. The resulting simulated sample datasets thus also have a target coverage of 100x or 1000x, respectively. Further details of the simulation can also be found in the evaluation workflow at https://github.com/orthanq/orthanq-evaluation-virus/blob/main/workflow/rules/simulation.smk.

#### 5 virus mix

The second considered dataset was obtained from an *in vitro* mixture of five different HIV-1 virus variants, HXB2, 89.6, JR-CSF, NL4-3, and YU-2 [19]. A library was generated from this mixture by whole-genome amplicon sequencing, with primer pairs for five amplicons that each span approximately 2500 bp and with neighboring amplicons overlapping by about 500 bp. Amplicons were then enzymatically fragmented and sequenced as paired-end reads to a length of 250 bp on an Illumina MiSeq. Since the mixture was controlled *in vitro* and evaluated in the original publication, ground truth fraction estimates of the five virus variants are available (Giallonardo et al. [19], Supplementary Tables 1 and 2).

#### SARS-CoV-2 co-infection datasets

The third dataset consists of 13 real SARS-CoV-2 patient samples from two studies. They all come from known and thoroughly characterized co-infections.

The first study is by Bolze et al. [4], in which a co-circulation of Delta and Omicron variants between November 2021 and February 2022 in the USA was studied. In this study, about 30k samples were screened and 18 co-infections were identified. Out of these 18 samples, we used all the 5 samples that have publicly available data (hosted by NCBI Sequence Read Archive, SRA, under the project ID PRJNA804575). The samples were sequenced using the target-capture method with a hybrid selection strategy. For preprocessing, adapters were trimmed from reads using Fastp [22].

The second study, by Combes et al. [11], investigates co-infection cases of Delta and Omicron during the fifth wave of the pandemic in France. The screening for identification of co-infection cases was performed on 3831 respiratory samples. Unexpected mutation profiles suggested co-infections in seven patient samples (0.2%). For six of those patients the co-infections were confirmed by amplicon-based whole genome sequencing. We used the resulting eight sequencing samples from those six patients, including two different time points from two patients. This dataset is publicly available on SRA (PRJNA809680). The preprocessing of these samples involved primer trimming, which needed to be handled carefully, as two different types of primers were used (Arctic v3 and v4). Reads were aligned to the SARS-CoV-2 reference genome (NC_045512.2) using BWA [23], primers were clipped with *samtools ampliconclip* [24] and the reads were extracted using *samtools fastq* [24].

Both studies contain samples that were found to be infected by Delta and Omicron variants of SARS-CoV-2. They each provide expected ground truth fractions, but with considerable error margins. For that reason, the fractions of identified virus variants in the ground truth may not sum up to exactly 100 percent. In cases where the sum exceeds 100%, Fig. 3 was clamped on the y axis to not exceed 100%. Pangolin and Nextclade both provide single virus variant predictions, whereas both Orthanq and Kallisto quantify abundances. For comparison between the usually fine-grained predictions and the ground truth wich was given on the lineage level (delta, omicron), we considered predictions starting with "AY" and "B.1.617.2" as "delta", "BA" as "Omicron" and "X" as "recombinant".

## Results

### Evaluation

#### SARS-CoV-2 simulated virus variant mixture

The simulated dataset can be seen as a set of synthetic co-infection samples. We quantified SARS-CoV-2 virus variants on all simulated samples with Orthanq and Kallisto, while omitting Pangolin and Nextclade since they only predict a single virus variant.

**Fig. 2:**
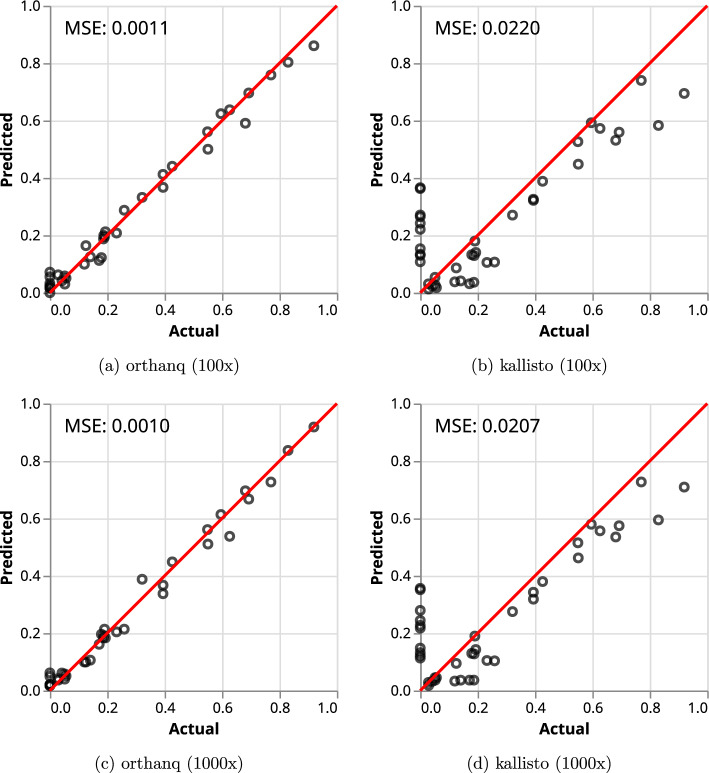
Orthanq and Kallisto predictions for the pandemics simulation dataset with 100$\times$ and 1000$\times$ coverages. ’Actual’ refers to fractions in the truth of the synthetic sample while ’Predicted’ refers to predicted fractions. Every prediction that has a hit in the truth is represented. Additional hits to nonmatching lineages are summed up to be represented as a single dot per sample. MSE: Mean Squared Error

It can be seen from Fig. 2 that Orthanq and Kallisto both manage to predict the simulated fractions quite accurately, while Orthanq’s predictions have a lower mean squared error. While Orthanq does not predict any haplotype that has not been simulated at a fraction greater than 0.1, Kallisto does so several times. Further, there seems to be a slight trend of underestimation for higher abundance haplotypes with Kallisto.

One sample from the dataset ("SimulatedSample1", which can be investigated in the Snakemake report under "Lineage Similarities") contains two virus variants HK.3 and EG.5.1.1, differing by only a single genomic variant. Orthanq accurately identifies and quantifies these two virus variants (see Supplementary Note 2). An independent simulated sample with two highly similar haplotypes BA.1.1.13 and BA.1.1.14, differing by three genomic variants and a distant Delta variant, B.1.617.2, is also predicted accurately with added fractions of 0.1 from BA.1.1.13, 0.2 from BA.1.1.14 and 0.7 from B.1.617.2 (see Supplementary Note 2). This shows that Orthanq is reliable for quantification of highly similar virus variants, even at low abundances.

#### Detection of co-infection

Second, we compare all tools on the samples with known co-infection by more than one SARS-CoV-2 virus variant at a time. For this purpose, we analyzed two publicly available datasets, five samples by Bolze et. al [4] and eight samples by Combes et al. [11].

**Fig. 3:**
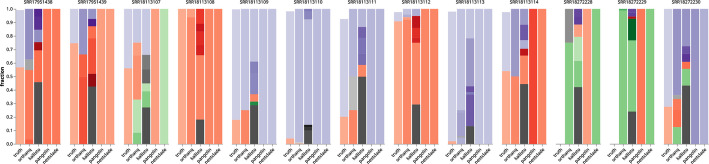
SARS-CoV-2 variant predictions for Orthanq, Kallisto, Pangolin and Nextclade for two studies that include 13 samples with co-infection by Delta and Omicron variants of SARS-CoV-2. Red color tones refer to ’Omicron’, ’purple’ to ’Delta’, green to ’recombinant’ and gray to other lineages. Mean squared error for orthanq predictions: 0.010, mean squared error for kallisto quantifications:0.051. (Error margins of the samples from PRJNA809680 are given as SRR18113107: Omicron: 56.1% ± 32.3%, Delta: 57.5% ± 36.6%; SRR18113108: Omicron: 100%, Delta: 0%; SRR18113109: Omicron: 17.9%± 5.2%, Delta: 81.8%± 6.1%; SRR18113110: Omicron: 4.1% ± 3.0%, Delta: 94.1% ± 3.7%; SRR18113111: Omicron: 20.2% ± 13.3%, Delta: 72.3% ± 12.5%; SRR18113112: Omicron: 90.8%± 5.7%, Delta: 6.8%± 3.7%; SRR18113113: Omicron: 2.1%± 3.7%, Delta: 96.0% ± 4.8%; SRR18113114: Omicron: 54.2%± 7.7%, Delta: 43.6% ± 11.5%)

Figure 3 shows that Orthanq performs superior for predicting fractions. It should be mentioned that the fractions given in the ground truth [11] are based on the computations of fraction estimations by taking into account genomic variant allele frequencies (VAFs) of a set of Omicron and Delta mutations. Hence, the ground truth resolution is at a high level only and does not provide data about individual virus variant fractions in the samples. For that reason, we can only perform comparisons on the level of Omicron vs. Delta. The predicted results of Orthanq and Kallisto in Fig. 3 are therefore more detailed than the truth, potentially showing additional variability within the virus population of the respective samples.

Another observation is that Pangolin and Nextclade always agree with the most abundant lineage prediction of Orthanq for the dominant variant, except for sample SRR18113107. Orthanq quantifies varying fractions of Omicron variant with a fraction of 0.4219, Delta variant with a fraction of 0.25 and recombinant variants with a total fraction of 0.3281. However, this sample has high error margins with fractions $56.1\% \pm 32.3\%$ for Omicron and $57.5\% \pm 36.6\%$ for Delta, which may explain the discrepancy. In the original study by Combes et al. [11], this sample (from patient P6) is also confirmed to be a product of recombination by omicron and delta, as validated by SMRT sequencing. Nextclade predicts "XD" as the dominant variant, while Orthanq predicts another recombinant, XS as a Delta-Omicron recombinant [25], with a fraction of 0.0917. Kallisto supports this finding by having XS with a fraction of 0.12 and XD [26] with a fraction of 0.06 in the sample.

Two samples, SRR18272228, SRR18272229 are recombinant samples that were also validated experimentally by orthogonal genotyping array and long read sequencing as mentioned by Bolze et al. [4], however without providing fractions or details beyond that the recombination is between delta and omicron. Concordantly, SRR18272229, referred to as RECOMB1 in the paper, is predicted to contain the recombinant "XS" [25] with 98.8% fraction by Orthanq. Kallisto supports this finding by containing mostly recombinant strains as majority. Likewise, Nextclade claims XS while Pangolin assigns Omicron (BA.1.1) for this sample. For the other recombinant SRR18272228, referred to as RECOMB2, Orthanq predicts 75% of XS and 25% of other (B). Kallisto quantifies about less than 50% recombinants, while the rest points to Omicron, Delta and other. Pangolin predicts Omicron (BA.1) while Nextclade predicts the recombinant XS for this sample.

The sample SRR18272230, also known as HMIX16, is predicted to have 63.75% of Delta, 20.77% of Omicron. While this already correlates with the numbers in the truth, Orthanq in addition predicts 12.46% of the recombinant XS. This finding is also in line with the findings of the original study, as there was evidence of a recombinant variant with read pairs separately supporting the presence of a Delta, an Omicron and a Delta-Omicron recombinant.

#### 5-virus-mix

**Fig. 4:**
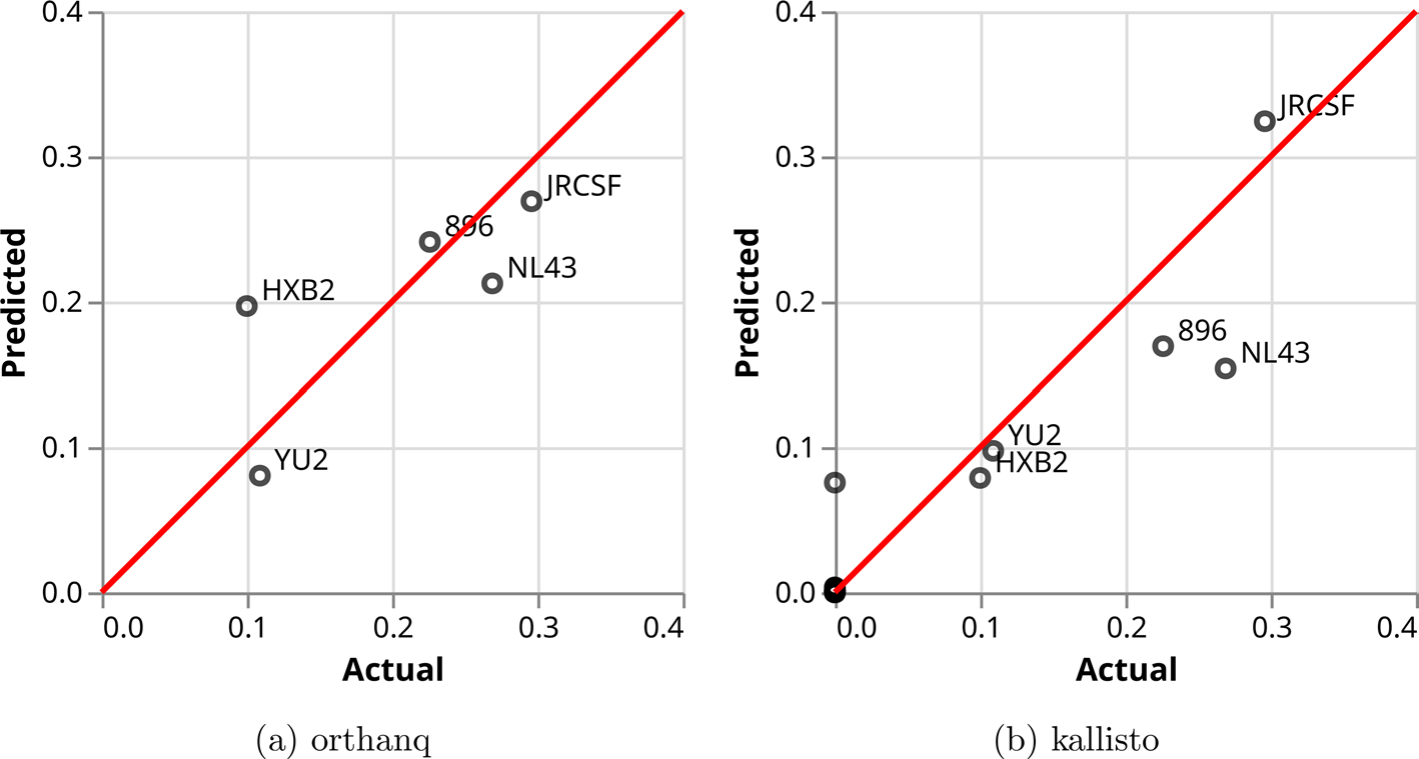
Orthanq and Kallisto predictions for the 5-virus-mix. Each circle represents one HIV strain. Predictions are better the closer they are to the red diagonal. Note that Kallisto predicts over 900 additional strains with a small, albeit non-zero, fraction (the dots at the origin)

For the 5-virus-mix dataset, Fig. 4 shows that Orthanq and Kallisto perform similarly, with their predicted fractions compared to the ground truth. However, Orthanq identifies only the five virus variants accurately out of 2833 virus variants and correctly determines that all other virus variants have a fraction of 0 in this sample. In contrast, Kallisto predicts over 900 additional variants with a small, albeit non-zero fraction, the most abundant of which is MK086129.1 with a fraction of 7%. The Orthanq prediction deviates most from the truth for HXB2. Kallisto deviates most for HXB2 and NL43. Orthanq quantifies HXB2 with a fraction of 0.1969, while the global (full length coding region) reconstruction and quantification by the original authors reports 0.1 ± 0.004 as its estimate (Giallonardo et al. [19], Supplementary Table 2). However, they also report very uneven coverage across the genome for the respective Illumina sequencing sample (Giallonardo et al. [19], Supplementary Figure 1). This uneven coverage seems to imply uneven coverage of individual virus variants across the genome, so they also did a gene-wise haplotype reconstruction and quantification (Giallonardo et al. [19], Supplementary Figure 3). With the gene-wise estimation, they report an HXB2 frequency estimate of 0.112 ±0.059 (Giallonardo et al. [19], Supplementary Table 1). This demonstrates a considerably larger uncertainty for the ground truth values, which is probably caused by differential amplification between the five amplicons and between the virus variants (for each of the amplicons).

### Transparent visualization and diagnostic Datavzrd reports

**Fig. 5:**
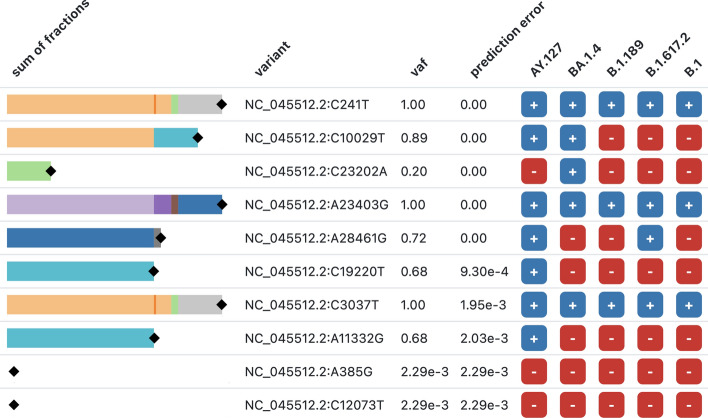
An example screenshot from the Datavzrd report generated by Orthanq. The columns that are the same in every report are as follows: ’sum of fractions’ refers to the sum of haplotype fractions for a contribution from the variant, ’vaf’ refers to the observed variant allele frequency (maximum a posteriori) and ’prediction error’ is the difference between the observed VAF and the VAF induced by summing the fractions. The rest of the columns indicates in which of the five haplotypes that are chosen by Orthanq’s prediction process each variant is present

Orthanq aims to make its decisions transparent by reporting detailed visualizations of the evidence underlying its predictions. Since Orthanq version 1.12, this now uses Datavzrd [27]. With Datavzrd, we can quickly and easily create interactive, visual, and portable reports of tabular data, requiring only a declarative configuration file and no programming. Figure 5 shows a screenshot from an example report for a SARS-CoV-2 coinfection sample. For each genomic variant, the report shows: (i) a stacked bar chart of the predicted haplotype fractions for haplotypes in which the genomic variant occurs (column ’sum of fractions’); (ii) the position and substitution of the variant (column ’variant’); (iii) the observed VAF (the fraction of the genomic variant allele among all alleles at the respective position as estimated by Varlociraptor [15]), shown as a black marker on top of the stacked bar chart and in the column ’vaf’; (iv) the difference between the VAF observed for this genomic variant (from column ’vaf’) and the sum of the predicted haplotype fractions that have this genomic variant (from column ’sum of fractions’; this is recorded in the column ’prediction error’); (v) in which haplotypes it occurs (remaining columns, one per candidate haplotype). One can search and filter by both genomic variants and haplotypes, enabling detailed investigations of individual pieces of evidence.

**Fig. 6:**
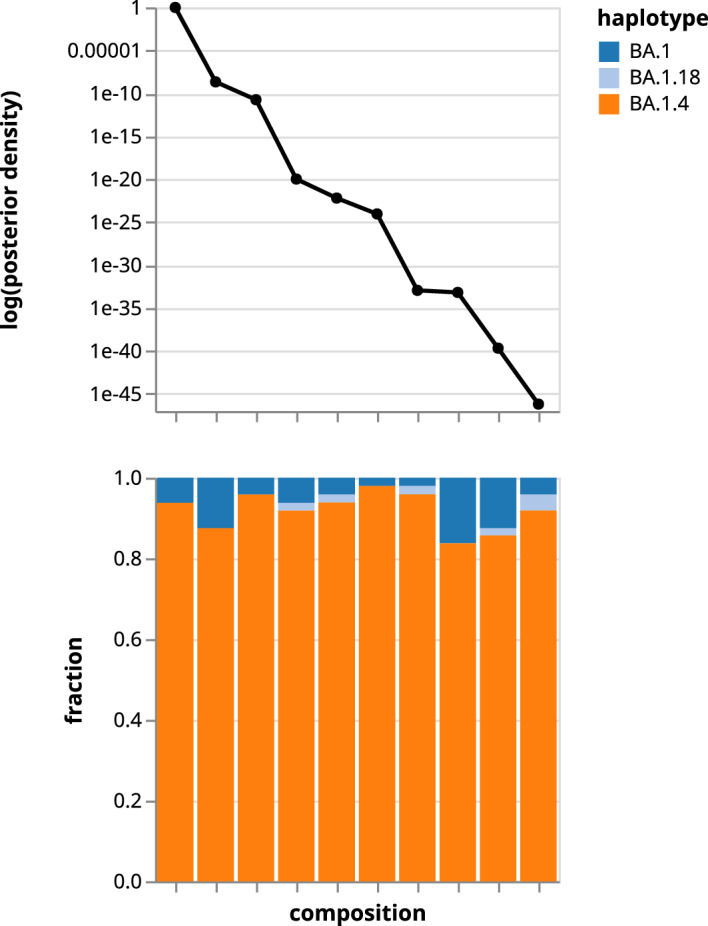
The solution plot of Orthanq showing the uncertainty in the prediction of SRR18113108. 10 most likely solutions/compositions are given together with computed posterior densities as well as fraction estimates

In addition, Fig. 6 shows Orthanq’s visualization of the 10 most likely virus variant frequency compositions in the sample. The plot captures Orthanq’s prediction (un-)certainty at a glance, by showing (i) how quick the drop-off in posterior density is from the most likely virus variant combination compared to the next likely combination, and (ii) how the fraction estimates of contained haplotypes differ between the most likely solutions.

**Fig. 7:**
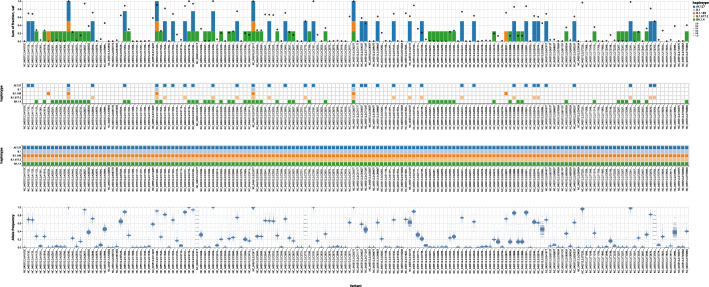
Excerpt from automatically generated ’best solution’ plot of Orthanq showing mode of operation of the Bayesian model for a SARS-CoV-2 coinfection sample. The top barchart displays the sum of fractions with bars for each predicted haplotype that is explained by the corresponding variant, circles represent observed variant allele frequency. Second and third dotplots represent genotype and coverage matrices, with the former referring to whether the haplotype contains the variant or not while the latter referring to whether the variant region is covered by the lineage or not. The last plot shows the allele frequency distribution of each variant with Phred probabilities and this is obtained from Varlociraptor

Figure 7 shows more details for the most likely solution for a combination of haplotype (i.e. virus variant) frequencies given by Orthanq, together with the evidence supporting it. It consists of four plots that share the x-axis: genomic variants ordered by reference sequence position. In the first plot (from the top), the contribution of each genomic variant to the haplotype fraction estimation is shown with stacked bars, one color for each haplotype (similar to the Datavzrd report in Fig. 5). Additionally, a point estimate of the genomic variant’s allele frequency is shown. In the second plot, the genotype matrix records the presence of genomic variants in each haplotype. With identical color coding for the haplotypes, we can easily determine the contribution of each genome variant from the fractions in the first plot right above. The third plot indicates which genomic variant positions are covered by a haplotype. If the nucleotide sequence stretch containing a genomic variant is missing in the FASTA reference sequence record of a lineage, this is shown as a white square in this plot. Finally, the bottom plot shows the allele frequency distribution of each genomic variant as provided by Varlociraptor [15], quantifying the uncertainty of the estimations at individual loci (also compare to the allele frequency point estimates from the first plot).

## Discussion

Orthanq performs uncertainty aware quantification of arbitrary haplotypes. While our first publication presented its use for HLA typing [14], this work demonstrates that Orthanq can also accurately quantify virus variants. The manuscript details improvements on the scalability and reliability of the method, as well as on the visualizations for assessing its results. We evaluated Orthanq in comparison with other solutions for virus variant identification and quantification, using a SARS-CoV2 pandemic simulation dataset, a real SARS-CoV2 co-infection dataset and an HIV-1 *in vitro* mixture dataset.

The results show that Orthanq reliably quantifies virus variant mixtures of varying fractions across different viral species. In doing so, Orthanq is consistently more accurate than the other evaluated tools across all three evaluation datasets. It especially reports fewer false positives than Kallisto, and correctly identifies recombinant samples in the co-infection dataset (unlike Pangolin). The analysis of the co-infection dataset further highlights a central advantage of using quantitative approaches like Orthanq or Kallisto: in contrast to Pangolin and Nextclade, they can determine mixtures of virus variants in the same sample and not only predict the most abundant virus variant from a single *de novo* assembly process.^6^ While the quantification of virus variants that are not previously known is currently not possible with Orthanq (or any of the tested tools), the analysis of the pandemic simulation dataset clearly shows that Orthanq reliably quantifies known virus variants, even at low abundances and with virus variants differing in as few as a single genomic variant. The HIV-1 mixture dataset demonstrates that Orthanq works independently of the considered virus.

In addition, the model and implementation of Orthanq is not limited to a particular type of sequencing data. The only requirement is for the data to cover all candidate genomic variants from all virus variants of a virus species under consideration. Hence, for any well-characterized virus species (implying a considerable number of known virus variants), this will require coverage of the whole genome for virus variant quantification.

Orthanq has two more points that set it apart from all the other approaches we compared against: First, Orthanq is the only generally applicable tool that is able to determine the number of virus variants in a sample. Other tools designed for virus variant identification will only determine the most prevalent virus variant. While Kallisto can be repurposed to quantify virus variants in a sample, it will assign non-zero fractions to virus variants that are not in the sample but share k-mers with a virus variant that is in the sample. Second, Orthanq does not only determine virus variants in a virus mixture, but also reports the uncertainty in the prediction, providing posterior densities of the reported haplotype compositions, as well as showing alternative solutions. With the diagnostic plots that Orthanq offers, users can investigate the model decisions and the underlying uncertainty in depth, including the newly added Datavzrd view for interactive exploration. In the best solution plot, users can even pinpoint individual discordant genomic variants; namely candidate genomic variants that clearly have non-zero allele frequencies, but are not from any of the virus variants chosen for the best solution. Such discordant variants might come from novel viral quasispecies that arose through mutations in the sampled infection that recapitulate a known genomic variant from another virus variant.

Finally, it should be highlighted that a single virus sample taken from a host individual does not suffice to distinguish whether the found virus variants are really co-infections or instead viral quasispecies that arose from infection by a single virus variant followed by subsequent mutation within the host. While this difference might often be of limited practical relevance (since the effect of having multiple virus variants in the host can be the same), it can be of interest from a research perspective. Here, Orthanq’s reliance on Varlociraptor becomes particularly advantageous. In case time series sequencing data is available for the virus infection of a host individual, the corresponding samples from different time points can be jointly considered by Varlociraptor (modeling in the same so-called scenario,^7^), if available even under consideration of prior knowledge about mutation rates. Then, Orthanq applied to each time point will consider the jointly determined allele frequencies of the set of candidate variants, providing uncertainty aware estimates of the virus variants found in the host across the time points. This will allow to see which virus variants have been there in the first place, and which arose later via mutations within the host.

## Conclusion

This paper extends and evaluates Orthanq for uncertainty aware virus variant quantification and presents methodological and technical improvements. We show that Orthanq outperforms other state of the art approaches for detecting virus variants in sequencing data, especially by also quantifying virus variants in co-infections. Leveraging the generic nature of Varlociraptor’s genomic variant calling model, Orthanq can work with any virus type and both second (short read) or third generation (long read) sequencing data. With extensive built-in visualizations and reporting of alternative solutions with posterior densities, users can easily evaluate the uncertainty of the results.

In future work, we will improve the reporting on genomic variants: on those that occur in addition to the predicted haplotypes, as well as on those where the observed VAF deviates from the VAF expected from the combination of predicted haplotypes. Moreover, we will investigate the applicability of Orthanq for wastewater monitoring, which is currently hampered by amplification biases causing less reliable VAF observations in such data.

## Supplementary Information

Supplementary file 1 (pdf 96 KB)

## Data Availability

The datasets analyzed in the study can be found on NCBI Sequence Read Archive under PRJNA809680, PRJNA804575 and PRJNA217811.

## Footnotes

1 We will only refer to virus species, strains or lineages throughout the manuscript, as appropriate in the respective context. However, for the purposes of our method, the same claims make sense across the species, strain, lineage or subtype level.

2 One important thing to note, is that such tools for virus variant identification cannot identify previously unknown virus variants. This contrasts with methods used for viral haplotype reconstruction, that are for example developed to pinpoint viral quasispecies. Such viral quasispecies can arise during rapidly evolving infections, when (multiple) mutations occur within a host. Tools for haplotype reconstruction perform a *de novo* assembly of reads into contigs. However, this comes with several drawbacks: (i) the *de novo* assembly can eliminate genuine variation [5], making it impossible to disambiguate very similar virus variants; (ii) resulting contigs often cover only parts of virus variant genomes, so that the assignment of contigs to known virus variants can remain ambiguous; (iii) the solved problem is computationally more challenging (no prior knowledge is used) such that in practice available methods can have issues with long virus genomes such as SARS-CoV-2. For virus identification, we thus focus on tools that rely on the prior knowledge of virus variant genome sequences; either directly, or by taking the extra step of placing assembled sequences within phylogenies of known virus variant sequences. Nevertheless, we provide a detailed assessment of haplotype reconstruction approaches in Supplementary Note 1.

3 It seemed plausible that tools using de novo assembly for viral haplotype (may also be referred to as viral quasispecies assembly methods) analyses might also be able to identify or even quantify virus variants. Therefore, we researched the most commonly used tools and attempted to run them on our evaluation datasets. However, none of them succeeded in clearly quantifying virus variants due to one or more or the following reasons: (i) the tool could not be installed; (ii) on one or more of our datasets, the tool threw an error that we could not debug in a reasonable timeframe; (iii) the tool only produces contigs, most of which do not cover full virus variant genomes and whose (post-hoc) assignment to virus variants remains ambiguous (in contrast to Pangolin and Nextclade, tools that explicitly incorporate this step). We document the tools we have tried and reasons for their exclusion in Supplementary File 1.

4 https://www.hiv.lanl.gov/content/sequence/NEWALIGN/align.html.

5 https://data.nextstrain.org/files/workflows/forecasts-ncov/gisaid/pango_lineages/usa.tsv.gz

6 Haplotype reconstruction or viral quasispecies assembly are related methods, in which the reads are also assembled into contigs. However, these contigs usually only cover parts of the virus genome and then have to be assigned to known virus variants. With *de novo* assembly errors and incomplete coverage of the genome, this process is not as straightforward as the quantification against known virus variants without any assembly procedure, which is indicated by the issues we had with such tools that are outlined in the supplementary.

7 https://varlociraptor.github.io/docs/calling/#generic-variant-calling.
