# Supplementary material for: Virus variant quantification with Orthanq
Source: BMC Bioinformatics. 2026 Feb 4;27:49. doi: 10.1186/s12859-026-06387-2 (PMC12930645; doi:10.1186/s12859-026-06387-2)
Supplement: Supplementary file 1 — (pdf 96 KB) [file 12859_2026_6387_MOESM1_ESM.pdf]

1 Supplementary Note 1: comparing against viral haplotype reconstruction approaches

Tools using de novo assembly for haplotype reconstruction could also be able to identify or even quantify virus variants, despite their slightly different aim: they assemble contigs but usually do not quantify contigs and do not assign them to known virus variants. In order to still attempt a comparison, we researched the most commonly used tools and tried to run them on our evaluation datasets.

However, none of them succeeded in clearly quantifying virus variants. This was due to one or more or the following reasons: (i) the tool could not be installed; (ii) on one or more of our datasets, the tool threw an error that we could not debug in a reasonable timeframe; (iii) the tool only produced partial contigs. Especially for points (ii) and (iii), we think that these are due to inherent properties of these methods: (a) as no prior knowledge is used, the problem to solve is computationally more challenging, and the methods will have resource issues with longer virus genomes such as SARS-CoV-2; (b) the *de novo* assembly can eliminate low frequency genuine variation, making it impossible to tell apart virus variants that differ in only one or a few variants; (c) the resulting contigs usually cover only parts of virus variant genomes, so that they cannot be assigned to known virus variants unambiguously; (d) even if contigs can be assigned to virus variants, they often don't come with abundance estimates, thus not allowing any quantification of the assigned virus variants.

We nevertheless tried to achieve virus variant quantification with a number of state of the art tools for viral haplotype reconstruction. Here, we document the tools we have tried, as well as the reasons for their exclusion from the comparisons in the main manuscript.

PredictHaplo [1] (`https://github.com/cbg-ethz/PredictHaplo`) reconstructs haplotype contigs from next generation sequencing (NGS) data. This tool provides abundance estimation of identified contigs.

We were able to install PredictHaplo via bioconda (`https://bioconda.github.io/recipes/predicthaplo/README.html`). However, the tool resulted in a segmentation fault error when run on a SARS-CoV-2 infected sample and a simulated sample. As, at the time of writing, multiple related error reports can be found as unresolved issues on GitHub (`https://github.com/cbg-ethz/PredictHaplo/issues/31`, `https://github.com/cbg-ethz/PredictHaplo/issues/28`), we concluded that this is not something we can debug in a reasonable timeframe. Thus, it was not possible to obtain contigs with PredictHaplo.

ShoRAH [2] (`https://github.com/cbg-ethz/shorah`) also reconstructs haplotype contigs from NGS data. This tool provides abundance estimation of identified contigs.

We were able to install ShoRAH via bioconda (`https://bioconda.github.io/recipes/shorah/README.html`). However, the tool resulted in a python index error when run on

a SARS-CoV-2 infected sample. We added our information to an (at the time of writing) unresolved issue reporting this problem: `https://github.com/cbg-ethz/shorah/issues/83` Thus, it was not possible to obtain contigs.

Qure [3] can reconstruct viral quasispecies. However, we did not find any installation or usage instructions, neither in the original manuscript, nor on its sourceforge page at https://sourceforge.net/projects/qure/. Thus, it was excluded from this evaluation.

Devider [4], is a tool used for haplotype reconstruction using long reads. As it is not applicable to the evaluation data used here, it was excluded from the evaluation.

Virus-vg [5] is a tool used for viral quasispecies reconstruction using NGS data. This tool provides abundance estimation of identified contigs. For the SARS-CoV-2 infected sample, the program crashes after exhausting large amounts of memory, and thus seems unsuitable for larger viral genomes.

On the 5-virus-mix, Virus-vg created 31 contigs, also providing a computed abundance for each. In order to assign the contigs to known virus variants, we mapped the contigs to the reference containing all HIV lineages. However, the contigs did not map to any lineage, hence we could not assign them to any lineages. Additionally, as mentioned in the paper by Baijens et al., 2019 we have also applied long terminal repeat (LTR) removal on the raw reads. However, we were not able to get results for this application due to a long computational runtime.

FC-virus (`https://github.com/qdu-bioinfo/FC-Virus`) is a tool used for reconstruction of full-length consensus sequences for viral quasispecies. This tool only outputs a single contig for a SARS-CoV-2 infected sample, which does not cover the full genome. In addition, it does not provide any abundance estimation. Thus, we could not consider it for our evaluation.

Haploclique [6] is a tool used for viral quasispecies reconstruction using NGS data. The tool provides abundance estimation. However, the tool has not been maintained for 7 years and we had to manually halt it after running for more than 72 hours on a single sample. Thus, an evaluation would take a prohibitive amount of time.

Spades [7] is a versatile tool used for assembling many kinds of sequencing data for bacteria and viruses. For the SARS-CoV-2 infected sample, it outputs 1800 contigs, and it does not provide any abundance estimation. Thus, it is not possible to reasonably compare it with virus variant quantification tools.

PEHaplo [8] is a tool used for haplotype reconstruction. It outputs identified contigs, but does not quantify them. Thus, it is not possible to reasonably compare it with virus variant quantification tools.

aBayesQR [9] is a tool used for viral quasispecies reconstruction from NGS data. But it does not provide abundance estimates, and resulted in a segmentation fault error when we tried to run it. Thus, it is not possible to compare it with virus variant quantification tools.

Vstrains [10] is a tool used for viral quasispecies reconstruction from NGS data. This tool works with the output from the tool Spades (Spades must be run without the ‘/–rnaviral‘ option) and ‘libgraph‘ should be ‘>=2.45‘ as the dependency. The tool outputs contigs of varying lengths, but it does not provide abundance estimation. Thus, it is not possible to reasonably compare it with virus variant quantification tools.

V-pipe [11] is a Snakemake workflow that enables viral haplotype reconstruction and provides abundance quantification. For the SARS-CoV-2 infected sample, it runs without any errors but it results in empty output. We reported this issue to the tool authors, but it is still in discussion at the time of writing: `https://github.com/cbg-ethz/V-pipe/issues/180` Thus, it was not possible to compare it with virus variant quantification tools at the time of writing.

2 Supplementary Note 2: distinguishing very similar virus variants

To evaluate how similar virus variants can be for Orthanq to still clearly distinguish and quantify them, we conducted two analyses:

First, we computed the pairwise distances between virus variants for each sample in the pandemics simulation dataset, by counting the number of genomic variants by which they differ. This is summarized as a datavzrd table with a bubble plot, which can be found in the online Snakemake report under the category ”Lineage similarities” at `https://koesterlab.github.io/orthanq-virus-paper-pandemic/report.html`. For example, the row for SimulatedSample1 shows that the two lineages HK.3 and EG.5.1.1 differ in only a single variant position (column ”Diff3”).

When we cross-reference this with the ”solutions plot” under ”Orthanq detailed solutions” entries for ”SimulatedSample1”, we can see that Orthanq quantifies them close to the expected frequencies of 0.12 and 0.19, respectively, 0.1 and 0.19 for 100x sample and 0.1 and 0.18 for 1000x sample.

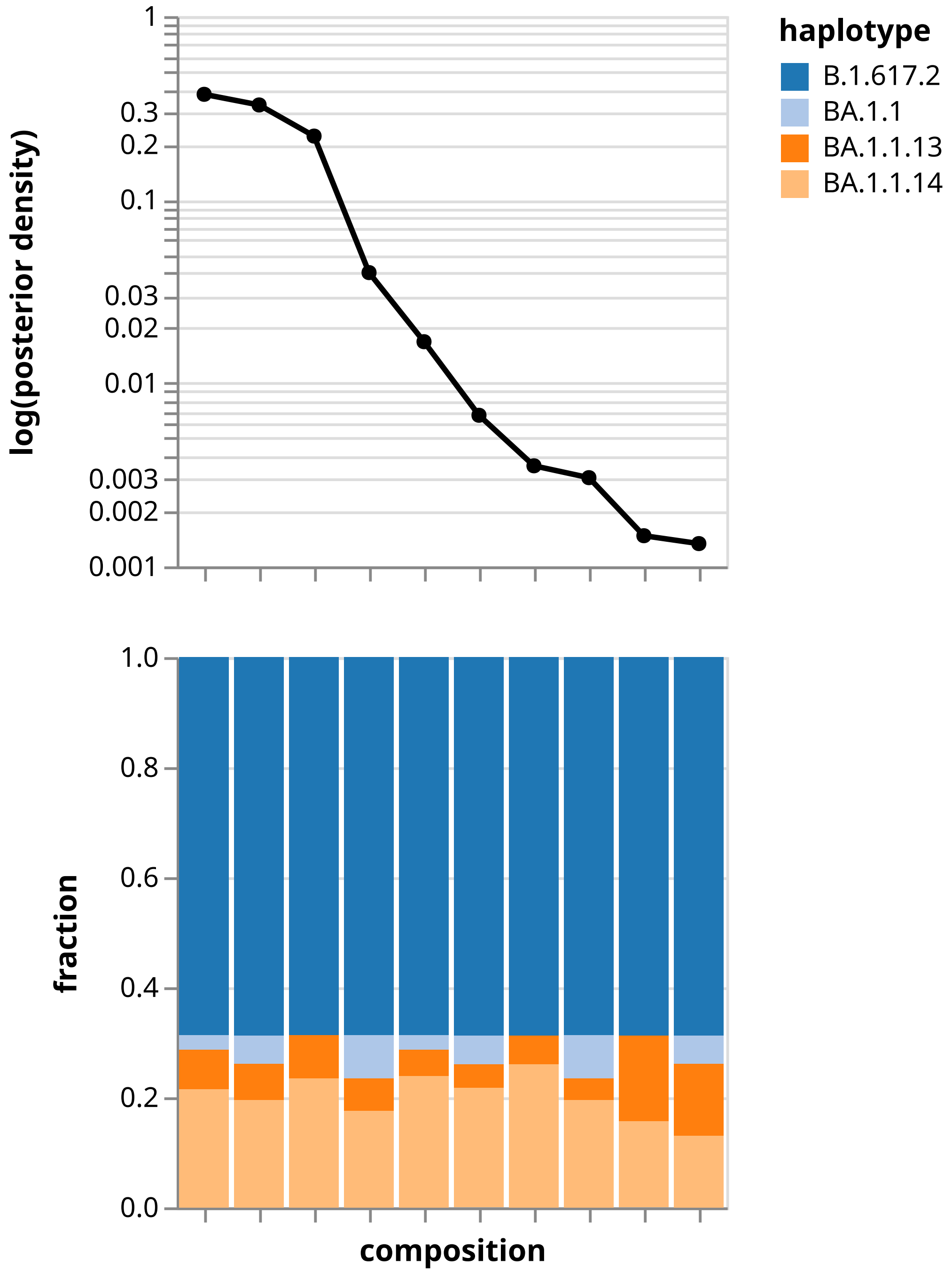

Figure 1: Solutions plot

Second, we further investigated how Orthanq performs for samples containing virus vari-

ants with high similarity, by simulating a dedicated sample at 100x. It contains the following three variants of SARS-CoV-2: two Omicron variants (BA.1.1.13 and BA.1.1.14) that differ in only three genomic variants (NC_045512.2:14107A>G, NC_045512.2:26078C>T and NC_045512.2:21C>T); and a distant Delta variant (B.1.617.2), differing by 69 variants from BA.1.1.13. We generated a synthetic sample with fractions of 0.1 from BA.1.1.13, 0.2 from BA.1.1.14 and 0.7 from B.1.617.2. Figure 1 gives the most likely solutions. Orthanq does not only identify the three virus variants out of 2950 candidate virus variants, but also estimates the correct abundances.

References

[1] Sandhya Prabhakaran, Melanie Rey, Osvaldo Zagordi, Niko Beerenwinkel, and Volker Roth. "HIV haplotype inference using a propagating dirichlet process mixture model". In: *IEEE/ACM transactions on computational biology and bioinformatics* 11.1 (2013), pp. 182–191.

[2] Osvaldo Zagordi, Arnab Bhattacharya, Nicholas Eriksson, and Niko Beerenwinkel. "ShoRAH: estimating the genetic diversity of a mixed sample from next-generation sequencing data". In: *BMC bioinformatics* 12.1 (2011), p. 119.

[3] Mattia CF Prosperi and Marco Salemi. "QuRe: software for viral quasispecies reconstruction from next-generation sequencing data". In: *Bioinformatics* 28.1 (2012), pp. 132–133.

[4] Jim Shaw, Christina Boucher, Yun William Yu, Noelle Noyes, and Heng Li. "devider: long-read reconstruction of many diverse haplotypes". In: *International Conference on Research in Computational Molecular Biology.* Springer. 2025, pp. 290–293.

[5] Jasmijn A Baaijens, Bastiaan Van der Roest, Johannes Köster, Leen Stougie, and Alexander Schönhuth. "Full-length de novo viral quasispecies assembly through variation graph construction". In: *Bioinformatics* 35.24 (2019), pp. 5086–5094.

[6] Armin Töpfer, Tobias Marschall, Rowena A Bull, Fabio Luciani, Alexander Schönhuth, and Niko Beerenwinkel. "Viral quasispecies assembly via maximal clique enumeration". In: *PLoS computational biology* 10.3 (2014), e1003515.

[7] Andrey Prjibelski, Dmitry Antipov, Dmitry Meleshko, Alla Lapidus, and Anton Korobeynikov. "Using SPAdes de novo assembler". In: *Current protocols in bioinformatics* 70.1 (2020), e102.

[8] Jiao Chen, Yingchao Zhao, and Yanni Sun. "De novo haplotype reconstruction in viral quasispecies using paired-end read guided path finding". In: *Bioinformatics* 34.17 (2018), pp. 2927–2935.

[9] Soyeon Ahn and Haris Vikalo. "aBayesQR: a Bayesian method for reconstruction of viral populations characterized by low diversity". In: *Journal of computational biology* 25.7 (2018), pp. 637–648.

[10] Runpeng Luo and Yu Lin. "Vstrains: De novo reconstruction of viral strains via iterative path extraction from assembly graphs". In: *International Conference on Research in Computational Molecular Biology*. Springer. 2023, pp. 3–20.

[11] Lara Fuhrmann, Kim Philipp Jablonski, Ivan Topolsky, Aashil A Batavia, Nico Borgsmüller, Pelin Icer Baykal, Matteo Carrara, Chaoran Chen, Arthur Dondi, Monica Dragan, et al. "V-pipe 3.0: a sustainable pipeline for within-sample viral genetic diversity estimation". In: *GigaScience* 13 (2024), giae065.
